# uEFS: An efficient and comprehensive ensemble-based feature selection methodology to select informative features

**DOI:** 10.1371/journal.pone.0202705

**Published:** 2018-08-28

**Authors:** Maqbool Ali, Syed Imran Ali, Dohyeong Kim, Taeho Hur, Jaehun Bang, Sungyoung Lee, Byeong Ho Kang, Maqbool Hussain

**Affiliations:** 1 Department of Computer Science and Engineering, Kyung Hee University, Yongin, Gyeonggi, Republic of Korea; 2 School of Engineering and ICT, University of Tasmania, Hobart, Tasmania, Australia; 3 Department of Software, Sejong University, Seoul, Gyeonggi, Republic of Korea; Jilin University, CHINA

## Abstract

Feature selection is considered to be one of the most critical methods for choosing appropriate features from a larger set of items. This task requires two basic steps: ranking and filtering. Of these, the former necessitates the ranking of all features, while the latter involves filtering out all irrelevant features based on some threshold value. In this regard, several feature selection methods with well-documented capabilities and limitations have already been proposed. Similarly, feature ranking is also nontrivial, as it requires the designation of an optimal cutoff value so as to properly select important features from a list of candidate features. However, the availability of a comprehensive feature ranking and a filtering approach, which alleviates the existing limitations and provides an efficient mechanism for achieving optimal results, is a major problem. Keeping in view these facts, we present an efficient and comprehensive univariate ensemble-based feature selection (uEFS) methodology to select informative features from an input dataset. For the uEFS methodology, we first propose a unified features scoring (UFS) algorithm to generate a final ranked list of features following a comprehensive evaluation of a feature set. For defining cutoff points to remove irrelevant features, we subsequently present a threshold value selection (TVS) algorithm to select a subset of features that are deemed important for the classifier construction. The uEFS methodology is evaluated using standard benchmark datasets. The extensive experimental results show that our proposed uEFS methodology provides competitive accuracy and achieved (1) on average around a 7% increase in f-measure, and (2) on average around a 5% increase in predictive accuracy as compared with state-of-the-art methods.

## Introduction

In the domain of data mining and machine learning, one of the most critical problems is the task of feature selection (FS), which pertains to the complexity of the appropriate choosing of features from a larger set of such [1]. FS performs a key role in the (so-called) process of “knowledge discovery” [2]. Traditionally, this task is performed manually by a human expert, thereby making it more expensive and time-consuming as compared with the use of an automatic FS, which has become necessary for the fast-paced digital world of today [3]. FS techniques are generally split into the three categories: of filter, wrapper, and hybrid, wherein each technique has capabilities and limitations [3–5]. Popular evaluation methods used for these techniques are *information-theoretic measures*, *correlational measures*, *consistency measures*, *distance-based measures*, and *classification/predictive accuracy*. A good FS algorithm can effectively filter out unimportant features [6]. Thus, in this regard, a significant amount of research has focused on proposing improved FS algorithms [7–11]; consequently, most of these algorithms use one or more of the aforementioned methods for performing FS. However, to date, there remains a lack of a comprehensive framework, which can select features from a given feature set. In order to design such a comprehensive FS methodology, the following two major technical issues must be solved:

1. How to rank the features without the use of any learning algorithm; high computational costs; and the presence of individual statistical biases of state-of-the-art, feature-ranking methods must be considered. In this case, the filter-based, feature-ranking approach is more suitable than the other two approaches (i.e., wrapper and hybrid). Filter-based methods evaluate a feature’s relevance in order to assess its usefulness without using any learning algorithm [1, 4]. Filter-based, feature-ranking methods are further split into two subcategories: univariate and multivariate. Univariate filter methods are simple and have high performance characteristics as compared with the other approaches [12]. However, even though the univariate filter-based methods are considered to be much faster and less computationally expensive than wrapper methods [4, 13], in reality, each method has its capabilities as well as its limitations. For example, information gain (IG) is a widely acceptable measure for ranking the features [14]; however, IG is biased towards choosing features with a large number of values [15]. Similarly, the chi-squared statistic determines the association between a feature and its target class, but is sensitive to sample size [15]. In addition, gain ratio and symmetrical uncertainty enhances the IG; however, both are biased towards features with fewer values [16]. Therefore, the designing an efficient feature-ranking approach and the overcoming of the aforementioned limitations compose our first goal.
2. Additionally, how to find a minimum threshold value for retaining important features irrespective of the characteristics of the dataset must be determined. In this case, for defining cutoff points for removing irrelevant features, a separated validation set and artificially generated features approaches are used [8]; however, it is not clear how to find the threshold for the features’ ranking [17, 18]. Research has shown that finding an optimal cutoff value to select important features from different datasets can be problematic [17] and existing methodologies [15, 18] required educated guesses to specify a minimum threshold value for retaining important features. Therefore, designing an empirical method to specify a minimum threshold value for retaining important features and overcoming the aforementioned limitations is our second target.

Keeping in view these two facts, we have proposed an efficient and comprehensive FS methodology, called univariate ensemble-based FS (uEFS), which includes two innovative algorithms, unified features scoring (UFS) and threshold value selection (TVS) and which allows for us to select informative features from a given dataset. This study is the extension as well as a detailed review of some of our previous work [19], which proposed a consensus methodology for appropriate FS in order to generate a useful feature subset for the FS task. The UFS algorithm generates a final ranked list of features after a comprehensive evaluation of a feature set without (1) using any learning algorithm, (2) high computational costs, and (3) the existence of any individual statistical biases of state-of-the-art, feature-ranking methods. The current version of the UFS has been plugged into a recently developed tool named the data-driven knowledge acquisition tool (DDKAT) [19] to assist the domain expert in selecting important features for the data preprocessing task. The DDKAT supports an end-to-end knowledge engineering process for generating production rules from a dataset [19]. The current version of the UFS code and its documentation are freely available and can be downloaded from the GitHub open source platform [20, 21]. Similarly, the TVS provides an empirical algorithm to specify a minimum threshold value for retaining important features irrespective of the characteristics of the dataset. It selects a subset of features that are deemed important for the classifier construction.

The motivation behind the uEFS is to design and develop an efficient FS methodology for evaluating a feature subset through different angles and to produce a useful reduced feature set. In order to accomplish this aim, this study was undertaken with the following objectives: (1) to design a comprehensive and flexible feature-ranking algorithm to compute the ranks without (a) using any learning algorithm; (b) high computational costs; and (c) any individual statistical biases of state-of-the-art, feature-ranking methods and (2) to identify an appropriate cutoff value for the threshold to select a subset of features irrespective of the characteristics of the dataset with reasonable predictive accuracy.

The key contributions of this research are as follows:

1. The presentation of a flexible approach, called UFS for incorporating state-of-the-art univariate filter measures for feature-ranking
2. The proposal of an efficient approach, called TVS, for selecting a cutoff value for the threshold in order to select a subset of features
3. The demonstration of a proof-of-concept for the aforementioned techniques, after performing extensive experimentation which achieved (1) on average a 7% increase in the f-measure as compared with the baseline approach, and (2) on average a 5% increase in predictive accuracy as compared with state-of-the-art methods.

## Related works

This section briefly describes various existing studies related to the FS methodologies to filter out the irrelevant features. This study focused on presenting a comprehensive and flexible FS methodology based on an ensemble of univariate filter measures for the classifier construction. The following includes some relevant FS studies, which contain research surveys and ensemble-based approaches for ranking of features as well as identifying a cutoff value for the threshold in the domain of FS. Lastly, the overall perspectives of literature reviewed are presented.

A review of applied FS methods for microarray datasets was performed by Bolón et al. [22]. Microarray data classification is a difficult task due to its high dimension and small sample sizes. Therefore, FS is considered the de facto standard in this area [22]. Belanche and Gonzalez [7] studied the performance of different existing FS algorithms. A scoring measure was also introduced to score the output of FS methods, which was assumed as an optimal solution. To automate the FS, Liu and Yu [23] proposed a framework, which provided an important infrastructure to integrate different FS methods based on their common traits. Chen et al. [24] performed a survey on FS algorithms for an intrusion detection system. Experiments were performed for different FS methods i.e., filter, wrapper, and hybrid. Since the present study was not focused on comprehensible classifiers, it did not study the effects of FS algorithms on the comprehensibility of a classifier. In addition to this, no unifying methodology was proposed that was capable of categorizing existing FS methods based on their common characteristics or their effects on classifiers.

Regarding ensemble-based, feature ranking studies, Rokach et al. [9] and Jong et al. [10] examined the available ensemble-based, feature-ranking approaches to show the improvement in steadiness of FS. Similarly, Slavkov et al. [11] investigated numerous aggregation approaches of feature ranking and observed that aggregating feature rankings produced better results as compared with using the single feature-ranking method. In addition, Prati [8] also obtained better results using an ensemble feature-ranking approach. In the literature, a hybrid approach by combining the filter and wrapper methods was also presented that is able to eliminate unwanted features by employing a ranking technique [25]. A similar concept to an EFS approach has also been mentioned previously [2, 26]. For ensemble feature ranking, two aggregate functions called arithmetic mean and arithmetic median, respectively, were used to rank features [27]. Authors obtained the ranking by arranging the features from the lowest to the highest. Investigators assigned rank 1 to a feature with the lowest feature index and rank M to a feature with the highest feature index [27]. Similarly, other researchers aggregated several feature rankings to demonstrate the robustness of ensemble feature ranking that surges with the ensemble size [10]. Onan and Korukoğlu [12] presented an ensemble-based FS approach, wherein different ranking lists obtained from various FS methods were aggregated. They used a genetic algorithm to produce an aggregate-ranked list, which is a relatively more expensive technique than a weighted aggregate technique. The authors performed experiments of binary class problems, and it was not clear how the proposed method would deal with more complex datasets. Popular filter methods used for the ensemble-based FS approach include IG, gain ratio, chi-squared, symmetric uncertainty, one rule (OneR), and ReliefF. Most of the FS methodologies use three or more of the aforementioned methods for performing FS [1, 8, 15, 18, 27, 28].

With respect to identifying an appropriate cutoff value for the threshold, Sadeghi and Beigy [29] proposed a heterogeneous ensemble-based methodology for feature ranking. These authors used the genetic algorithm to determine the threshold value; however, a *θ* value is required to start the process. Moreover, the user is given an additional task of defining the notion of relevancy and redundancy of a feature. Osanaiye et al. [18] combined the output of various filter methods; however, a fixed threshold value i.e. one-third of a feature set, is defined a priori, irrespective of the characteristics of the dataset. Sarkar et al. [15] proposed a technique that aggregates the consensus properties of IG, chi-squared, and symmetric uncertainty FS methods to develop an optimal solution; however, this technique is not comprehensive enough to provide a final subset of features. Hence, a domain expert would still need to make an educated guess regarding the final subset. For defining cutoff points to remove irrelevant features, a separated validation set and artificially generated features approaches can be used [8], though it is not clear how to find the threshold for the features’ ranking [17, 18]. Finding an optimal cutoff value to use in selecting important features from different datasets is problematic [17].

Taking into consideration the aforementioned discussion, a significant amount of research [7–12, 15, 18, 24, 29] has focused on proposing improved FS methodologies; however, not so much consideration has been paid regarding selecting features from a given feature set in a comprehensive manner. These methodologies either used relatively more expensive techniques to select features or required an educated guess to specify a minimum threshold value for retaining important features.

## Materials and methods

This section first explains the process of uEFS methodology. Second, the UFS algorithm is explained through algorithms. Third, the TVS algorithm is presented and, lastly, the statistical measures, used for evaluating the performance of the proposed uEFS methodology, are explained.

### Univariate ensemble-based features selection methodology

In the FS process, normally, two steps are required [17]. In the first step, features are typically ranked, whereas, in the second step, a cutoff point is defined to select important features and to filter out the irrelevant features for building more robust machine learning models. In this regard, the proposed UFS algorithm [19] covers the first step of FS, while the TVS algorithm covers the second step.

**Fig 1:**
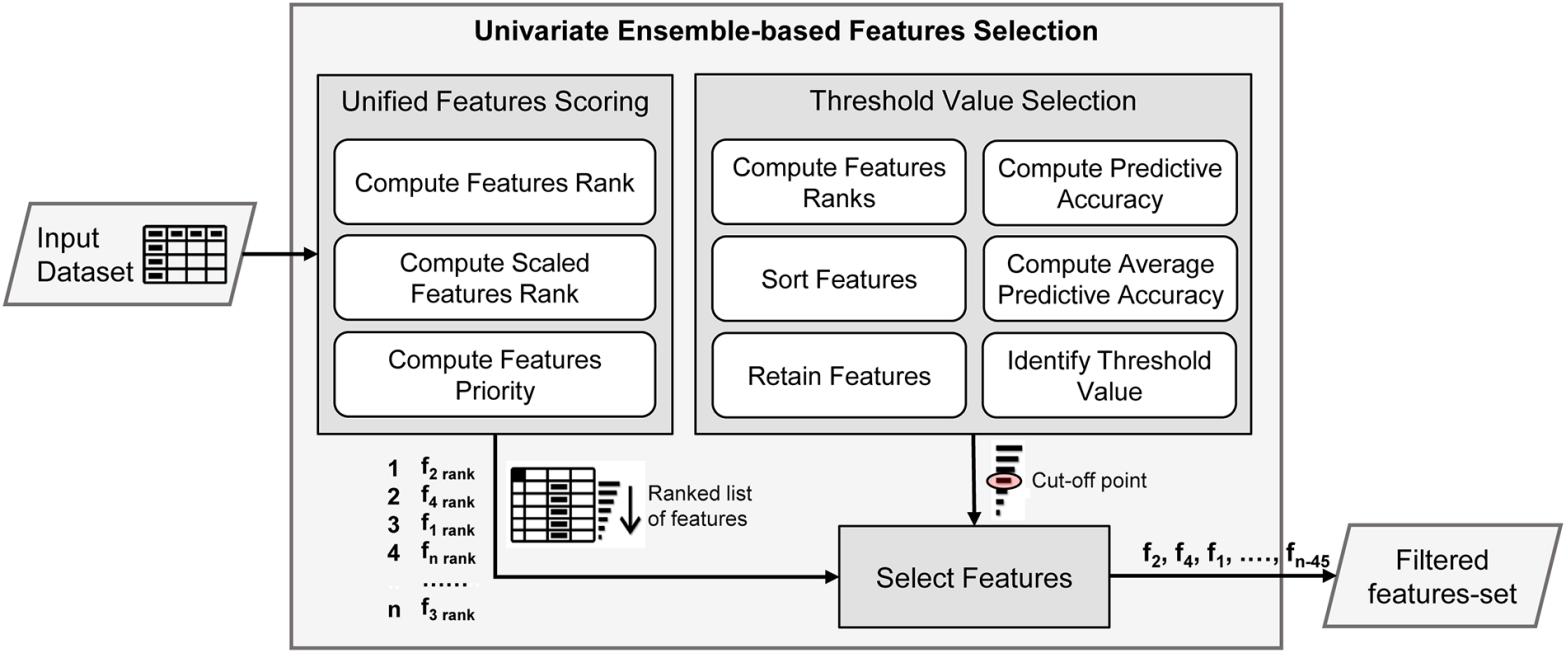
uEFS methodology.

Fig 1 shows the functional details of the proposed uEFS methodology, which consists of three major components of *UFS*, *TVS*, and *select features*. The *UFS* component evaluates the feature-set in a comprehensive manner and generates a final ranked list of features. For example, feature *f*_2_ has the highest priority, then feature *f*_4_, and so on, as shown in Fig 1. Similarly, the *TVS* component defines a cutoff point for selecting important features. Finally, the *select features* component filters out the irrelevant features from the final-ranked list of features based on a cutoff point and selects a subset of features that are deemed as important for the classifier construction. For example, *f*_2_, *f*_4_, *f*_1_, …, *f*_*n*−45_ is the list of features that were selected by the proposed uEFS methodology, as shown in Fig 1.

### Unified features scoring

UFS is an innovative feature ranking algorithm that tries to unify various filter-based methods [19] for the purpose of obtaining the final-ranked list of features. In this algorithm, univariate filter measures are employed to assess the usefulness of a selected feature subset in a multidimensional manner. These measures are better suited to high-dimensional datasets and provide better generalization [4, 13]. The UFS algorithm uses the *ensemble FS* (EFS) approach, which has been examined recently by some researchers [2, 26]. The EFS, an concept of ensemble learning, obtains a ranked list of features by incorporating the outcomes of different feature-ranking techniques [1, 27]. Generally, the intention of the EFS approach is to give an improved estimation to the most favorable subset of features for improving classification performance [2, 27, 30, 31]. As mentioned elsewhere [27], fewer studies have focused on the EFS approach to enrich the FS itself. Although ensemble-based methodologies have additional computational costs, these costs are affordable due to offering an advisable framework [32]. As discussed previously [27], there are three types of filter approaches: *ranking*, *subset evaluation*, and a *new FS framework* that decouples the redundancy analysis from relevance analysis. The UFS uses a *ranking* approach, as it is considered an attractive approach due to its simplicity, scalability, and good empirical success [27, 33]. Feature ranking measures the relevancy of the features (i.e., independent attributes) by their correlations to the class (i.e., dependent attribute) and ranks independent attributes according to their degrees of relevance [1]. These values may reveal different relative scales. To neutralize the effect of different relative scales, the UFS rescales the values to the same range (i.e., between 0 and 1) to make it scale-insensitive. For rescaling, the UFS allocates rank 1 to a feature with the highest feature index, as opposed to research that has been done previously [27], which assigned rank 0 to a feature having the topmost feature index. Following that, the UFS orders all scaled ranks in an ascending order and then aggregates them, as it is considered to be an effective technique [8]. The ordered-based, ranking-aggregation method combines the base rankings and considers only the ranks for ordering the attributes [8]. Finally, the UFS computes a mean value to compute weights and priorities of each feature.

UFS is described through Algorithm 1, which takes a dataset (i.e., *D*) as input and computes the ranks (scores) of the features after passing through key steps of the algorithm. UFS depends on *n* univariate filter-based measures, where the key rationale for *n* filter measures is to evaluate a feature through different considerations.

**Algorithm 1**: UFS (*D*)

 **Input**: *D*: Input data set (data)

 **Output**: *FR*− Features Ranks

1 *noOfAttrs* ← *numAttributes*(*data*)  // compute the number of attributes;

2  /* Consider *n* attribute evaluation measures, also called univariate filter measures (*AttrEv*_1_, *AttrEv*_2_, *AttrEv*_3_,…, *and AttrEv*_*n*_) */;

3  /* Compute the ranks using each selected measure */;

4 *CR*_1_[] ← *computeRanks*(*data*, *AttrEv*_1_) //where *CR* represents computed ranks;

5 *CR*_2_[] ← *computeRanks*(*data*, *AttrEv*_2_);

6 *CR*_3_[] ← *computeRanks*(*data*, *AttrEv*_3_);

7 *CR*_*n*_[] ← *computeRanks*(*data*, *AttrEv*_*n*_);

8  /* Compute the scaled ranks of each computed ranks using Algorithm 2 */;

9 *scaledRanks*_1_[] ← *scaleRanks*(*CR*_1_)   // invoke Algorithm 2;

10 *scaledRanks*_2_[] ← *scaleRanks*(*CR*_2_)  // invoke Algorithm 2;

11 *scaledRanks*_3_[] ← *scaleRanks*(*CR*_3_)  // invoke Algorithm 2;

12 *scaledRanks*_*n*_[] ← *scaleRanks*(*CR*_*n*_)  // invoke Algorithm 2;

13  /* Compute the combined sum of all computed ranks */;

14 *combinedranksSum* ← 0;

15 *combinedRanks*[];

16 **for** ∀ *noOfAttrs* ∈ *D* **do**

17  /* For each attribute, compute the combined rank by adding all computed scaled ranks */;

18  $combinedRanks_{i}\leftarrow \sum_{j=1}^{n}scaledRanks_{ji}$ //where n represents the number of filter measures;

19  *combinedranksSum* = *combinedranksSum* + *combinedRanks*_*i*_;

20 **end**

21  /* Rank the list in ascending order */;

22 *sortedRanks*[] ← *sort*(*combinedRanks*);

23  /* Compute the score, weight, and priority of each attribute */;

24 **for** ∀ *noOfAttrs* ∈ *D* **do**

25  *attrScores*_*i*_ ← *combinedRanks*_*i*_/*n* //where n represents number of filter measures;

26  *attrWeights*_*i*_ ← *combinedRanks*_*i*_/*combinedranksSum*;

27  *attrPriorities*_*i*_ ← *attributesScores*_*i*_ * *attributesWeights*_*i*_;

28   /* Assign an index (Rank ID) on ascending order to each attribute based on its priority value */;

29  *FR*[] ← *assignRank*(*attrPriorities*_*i*_);

30 **end**

31 **return** *FR*: *features ranks*

**Algorithm 2**: Scaling the Computed Ranks (*CR*)

 **Input**: *CR*: Input computed ranks (ranks)

 **Output**: *SR*− Scaled Ranks

1 *smallest* ← *ranks*_0_;

2 *largest* ← *ranks*_0_;

3 **for** ∀ *noOfAttrs* ∈ *CR* **do**

4  **if** *rank*_*i*_ > *largest* **then**

5   *largest* ← *rank*_*i*_;

6  **else**

7   **if** *rank*_*i*_ < *smallest* **then**

8    *smallest* ← *rank*_*i*_

9   **end**

10  **end**

11 **end**

12 *min* ← *smallest*;

13 *max* ← *largest*;

14 *SR*[] ← (*ranks* − *min*)/(*max* − *min*);

15 **return** *SR*: *scaled ranks*

In Algorithm 1, the first step is to compute the number of features from a given dataset. Then, in the second step, each feature in a dataset can be ranked using *n* number of univariate filter-based measures, as shown in Line 4 to Line 7 of Algorithm 1. After that, Algorithm 2 was used to scale (normalize) all computed ranks using the first filter measure. This step was repeated for the remaining (*n* − 1) measures as well as shown in Line 9 to Line 12. After the evaluation and scaling process, ranks aggregations were performed, as shown in Line 18 of Algorithm 1. Later, the comprehensive score as well as the weightage of each feature were computed, as shown in Line 25 and Line 26 of Algorithm 1. Finally, based on the contribution (i.e., individual measure score and relative weightage), a priority value of each feature was computed. This priority value of a feature was further utilized for ranking and feature subset selection.

**Fig 2:**
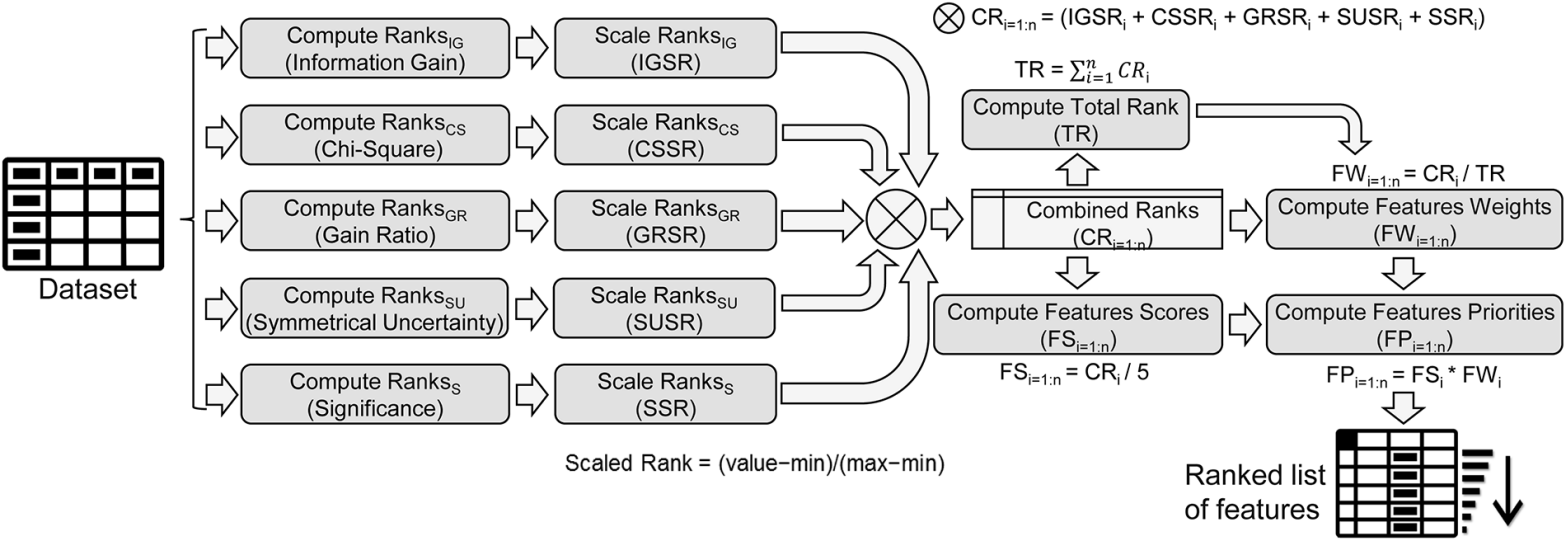
UFS algorithm [19].

For the proof-of-concept, five univariate filter-based measures—namely, IG, gain ratio, symmetric uncertainty, chi-squared, and significance [1, 8, 19, 27, 28]—were used to explain the process of the proposed unified features scoring algorithm. The reasons for selecting these five measures are described elsewhere [19]. Using these five filter measures, the process of the UFS is depicted in Fig 2. This process is also explained through an example.

### Threshold value selection

The process of FS starts once features are ranked. In order to select a subset of features, the TVS algorithm is introduced, which provides an empirical approach of specifying a minimum threshold value. Those attributes that score less than the minimum threshold value can be discarded for building more robust machine learning models. The proposed algorithm is implemented in Java language using WEKA API.

TVS is explained through Algorithm 3. This algorithm takes *n* datasets (i.e., *D*) and *m* classifiers (i.e., *C*) as input and sequentially passes them through mandatory steps of the algorithm to find the cutoff value from a predictive accuracy graph.

**Algorithm 3**: TVS (*D*, *C*)

 **Input**: *D* − (*d*_1_, *d*_2_,…,*d*_*n*_)  // *set of n datasets with varying complexities*

    *C* − (*c*_1_, *c*_2_,…,*c*_*m*_)  // *set of m machine learning classifiers*

 **Output**: *V* − *cutoff value*

1 initialization;

2 **for** *d*_*i*_ ← ***in*** *D* **do**

3  *d*_*i*_ ← *computeFeatureRank*(*d*_*i*_)  // rank each feature;

4  *d*_*i*_ ← *sortByRankASC*(*d*_*i*_)  // sort features by rank in ASC;

5 **end**

6 *P* ← 100;

7 **for** *d*_*i*_ ← ***in*** *D* **do**

8  **while** *P* ≥ 5 **do**

9    *k* ← *sizeOf*(*d*_*i*_) * (*p*/100)  // compute partition size;

10    *Acc* ← *newSet*()   // initialize empty set;

11    **for** *c*_*i*_ ← ***in*** *C* **do**;

12     *P*_*acc*_ ← *predictiveAccuracy*(*c*_*i*_, *topKFeatures*(*d*_*i*_, *k*));

13     *Acc*.*add*(*P*_*acc*_)   // add accuracy to set;

14    **end**

15    *AVG*_*acc*_ ← *computeAVG*(*Acc*)   // compute average accuracy;

16    *G* ← *Plot*(*AVG*_*acc*_, *k*)   // plot the average point;

17    *P* ← *P* − 5   // decrease the partition size by 5;

18  **end**

19 **end**

20 *V* ← *getCutoffValue*(*G*);

In Algorithm 3, first consider the *n* number of benchmark datasets having varying complexities. After that, compute the feature ranks using a ranker search mechanism and then sort them in an ascending order, as shown in Line 3 and Line 4 of Algorithm 3. Then, partition each dataset into different chunks (filtered datasets) from 100% to 5% features retained. Once filtered datasets are created, then consider *m* number of classifiers from various classifiers categories/families having varying characteristics (where *m* ≪ *n*) and feed each filtered dataset to these classifiers as shown in Line 6 and Line 11 of Algorithm 3. Following this, record predictive accuracies of these classifiers to each chunk of dataset partitioning using 10-fold cross validation approach (Line 12). Later, compute the average predictive accuracy of all classifiers as well as datasets against each chunk of dataset partitioning (Line 15). Finally, plot all computed average predictive accuracies against each chunk of dataset partitioning (Line 16) and identify the cutoff value from the plotted graph (Line 20).

**Fig 3:**
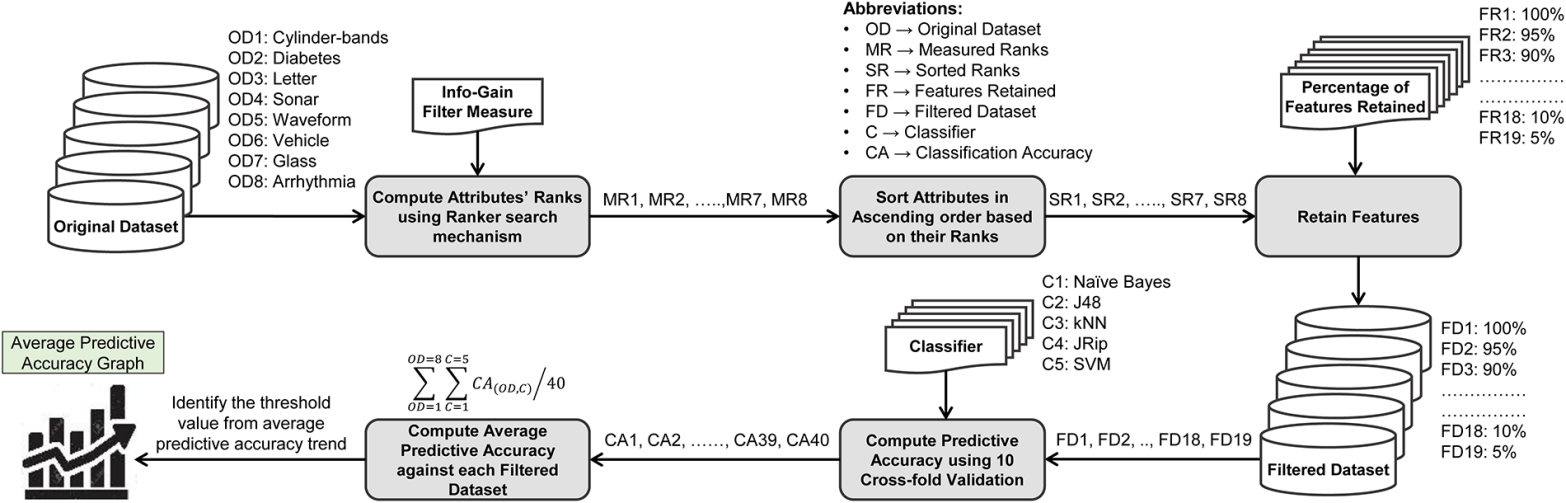
TVS algorithm.

For the proof-of-concept, eight datasets of varying complexities were used to explain the process of the proposed threshold selection algorithm. The process of threshold value selection is depicted in Fig 3.

**Fig 4:**
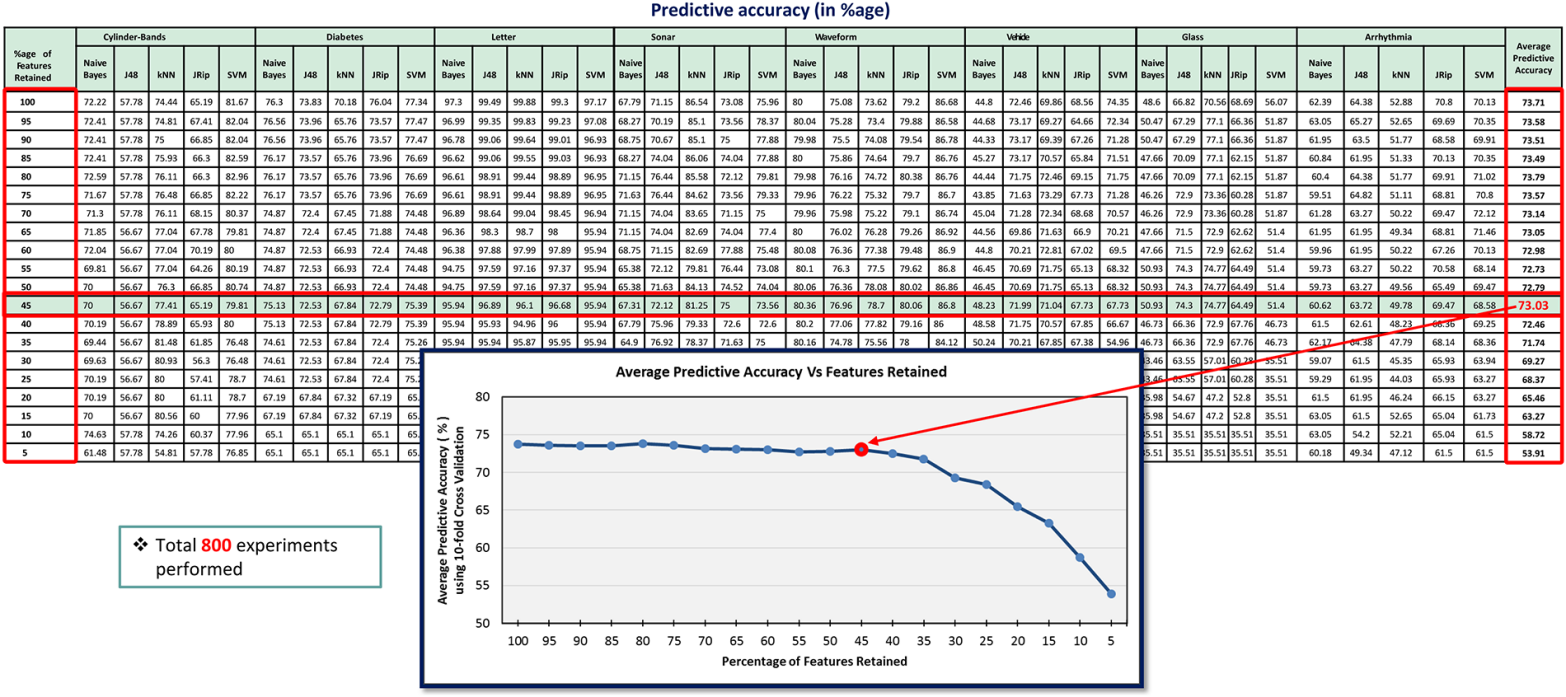
An average predictive accuracy graph using the 10-fold cross-validation technique for threshold value identification.

As depicted in Fig 3, each dataset (*Cylinder-bands*, *Diabetes*, *Letter*, *Sonar*, *Waveform*, *Vehicle*, *Glass*, *Arrhythmia*) was fed to the *IG* filter measure for computing attributes’ ranks. Then, all measured ranks of attributes of each dataset were sorted in ascending order. Afterwards, each dataset was partitioned into different chunks (filtered datasets) from 100% to 5% features retained, e.g., in case of an 80% chunk, the dataset retains nearly 80% of the highly ranked features, while 20% of the features, which are below the rank, are discarded. Each filtered dataset was fed to five well-known classifiers from various classifier categories/families having varying characteristics [e.g., naive Bayes from the *Bayes* category, J48 from the *Trees* category, k-nearest neighbors (kNN) from the *Lazy* category, JRip from the *Rules* category, and support vector machine (SVM) from the *Functions* category] and, using a *10-fold cross-validation* approach [8], predictive accuracies of these classifiers were recorded to each chunk of dataset partitioning, as illustrated in Table 1. Finally, an average predictive accuracy of all classifiers as well as the datasets against each chunk of dataset partitioning were computed. The main intuition of this process is to identify an appropriate chunk value that provides reasonable predictive accuracy and considerably reduces the dataset as well. Through empirical evaluation, it was found that a 45% chunk provided a reasonable threshold value of feature subset selection (Fig 4).

**Table 1:** Predictive accuracy (in %age) of classifiers using benchmark datasets.

%age of Features Retained	Naive Bayes	J48	kNN	JRip	SVM	Naive Bayes	J48	kNN	JRip	SVM	Naive Bayes	J48	kNN	JRip	SVM
	**Cylinder-Bands**					**Diabetes**					**Letter**				
**100**	72.22	57.78	74.44	65.19	81.67	76.3	73.83	70.18	76.04	77.34	97.3	99.49	99.88	99.3	97.17
**95**	72.41	57.78	74.81	67.41	82.04	76.56	73.96	65.76	73.57	77.47	96.99	99.35	99.83	99.23	97.08
**90**	72.41	57.78	75	66.85	82.04	76.56	73.96	65.76	73.57	77.47	96.78	99.06	99.64	99.01	96.93
**85**	72.41	57.78	75.93	66.3	82.59	76.17	73.57	65.76	73.96	76.69	96.62	99.06	99.55	99.03	96.93
**80**	72.59	57.78	76.11	66.3	82.96	76.17	73.57	65.76	73.96	76.69	96.61	98.91	99.44	98.89	96.95
**75**	71.67	57.78	76.48	66.85	82.22	76.17	73.57	65.76	73.96	76.69	96.61	98.91	99.44	98.89	96.95
**70**	71.3	57.78	76.11	68.15	80.37	74.87	72.4	67.45	71.88	74.48	96.89	98.64	99.04	98.45	96.94
**65**	71.85	56.67	77.04	67.78	79.81	74.87	72.4	67.45	71.88	74.48	96.36	98.3	98.7	98	95.94
**60**	72.04	56.67	77.04	70.19	80	74.87	72.53	66.93	72.4	74.48	96.38	97.88	97.99	97.89	95.94
**55**	69.81	56.67	77.04	64.26	80.19	74.87	72.53	66.93	72.4	74.48	94.75	97.59	97.16	97.37	95.94
**50**	70	56.67	76.3	66.85	80.74	74.87	72.53	66.93	72.4	74.48	94.75	97.59	97.16	97.37	95.94
**45**	70	56.67	77.41	65.19	79.81	75.13	72.53	67.84	72.79	75.39	95.94	96.89	96.1	96.68	95.94
**40**	70.19	56.67	78.89	65.93	80	75.13	72.53	67.84	72.79	75.39	95.94	95.93	94.96	96	95.94
**35**	69.44	56.67	81.48	61.85	76.48	74.61	72.53	67.84	72.4	75.26	95.94	95.94	95.87	95.95	95.94
**30**	69.63	56.67	80.93	56.3	76.48	74.61	72.53	67.84	72.4	75.26	95.94	95.94	95.92	95.94	95.94
**25**	70.19	56.67	80	57.41	78.7	74.61	72.53	67.84	72.4	75.26	95.94	95.94	95.92	95.94	95.94
**20**	70.19	56.67	80	61.11	78.7	67.19	67.84	67.32	67.19	65.1	95.94	95.94	95.99	95.94	95.94
**15**	70	56.67	80.56	60	77.96	67.19	67.84	67.32	67.19	65.1	95.94	95.94	95.94	95.94	95.94
**10**	74.63	57.78	74.26	60.37	77.96	65.1	65.1	65.1	65.1	65.1	95.94	95.94	95.94	95.94	95.94
**5**	61.48	57.78	54.81	57.78	76.85	65.1	65.1	65.1	65.1	65.1	95.94	95.94	95.94	95.94	95.94
	**Sonar**					**Waveform**					**Vehicle**				
**100**	67.79	71.15	86.54	73.08	75.96	80	75.08	73.62	79.2	86.68	44.8	72.46	69.86	68.56	74.35
**95**	68.27	70.19	85.1	73.56	78.37	80.04	75.28	73.4	79.88	86.58	44.68	73.17	69.27	64.66	72.34
**90**	68.75	70.67	85.1	75	77.88	79.98	75.5	74.08	79.54	86.78	44.33	73.17	69.39	67.26	71.28
**85**	68.27	74.04	86.06	74.04	77.88	80	75.86	74.64	79.7	86.76	45.27	73.17	70.57	65.84	71.51
**80**	71.15	76.44	85.58	72.12	79.81	79.98	76.16	74.72	80.38	86.76	44.44	71.75	72.46	69.15	71.75
**75**	71.63	76.44	84.62	73.56	79.33	79.96	76.22	75.32	79.7	86.7	43.85	71.63	73.29	67.73	71.28
**70**	71.15	74.04	83.65	71.15	75	79.96	75.98	75.22	79.1	86.74	45.04	71.28	72.34	68.68	70.57
**65**	71.15	74.04	82.69	74.04	77.4	80	76.02	76.28	79.26	86.92	44.56	69.86	71.63	66.9	70.21
**60**	68.75	71.15	82.69	77.88	75.48	80.08	76.36	77.38	79.48	86.9	44.8	70.21	72.81	67.02	69.5
**55**	65.38	72.12	79.81	76.44	73.08	80.1	76.3	77.5	79.62	86.8	46.45	70.69	71.75	65.13	68.32
**50**	65.38	71.63	84.13	74.52	74.04	80.06	76.36	78.08	80.02	86.86	46.45	70.69	71.75	65.13	68.32
**45**	67.31	72.12	81.25	75	73.56	80.36	76.96	78.7	80.06	86.8	48.23	71.99	71.04	67.73	67.73
**40**	67.79	75.96	79.33	72.6	72.6	80.2	77.06	77.82	79.16	86	48.58	71.75	70.57	67.85	66.67
**35**	64.9	76.92	78.37	71.63	75	80.16	74.78	75.56	78	84.12	50.24	70.21	67.85	67.38	54.96
**30**	64.42	71.15	80.29	73.08	72.12	80.12	74.74	73.22	77.2	83.24	46.81	61.7	63.83	60.64	50.47
**25**	62.98	70.67	73.56	69.23	73.56	75.24	72.92	69.62	74.42	79.86	44.92	61.58	61.58	57.68	47.52
**20**	63.46	71.63	69.23	71.15	74.52	66.3	64.62	58.28	66.82	70.52	43.85	57.33	53.31	54.49	46.57
**15**	58.65	69.23	64.9	66.83	69.23	59.14	57.58	51.32	57.42	61.22	41.49	50.12	49.29	42.08	42.55
**10**	56.73	62.02	57.69	57.69	58.17	51.78	50.42	42.28	48.54	51.78	40.07	43.62	40.9	32.62	30.85
**5**	55.29	50.48	53.85	54.33	56.73	39.02	38.56	34.44	36.06	38.38	25.65	25.65	25.65	25.65	25.65
	**Glass**					**Arrhythmia**									
**100**	48.6	66.82	70.56	68.69	56.07	62.39	64.38	52.88	70.8	70.13					
**95**	50.47	67.29	77.1	66.36	51.87	63.05	65.27	52.65	69.69	70.35					
**90**	50.47	67.29	77.1	66.36	51.87	61.95	63.5	51.77	68.58	69.91					
**85**	47.66	70.09	77.1	62.15	51.87	60.84	61.95	51.33	70.13	70.35					
**80**	47.66	70.09	77.1	62.15	51.87	60.4	64.38	51.77	69.91	71.02					
**75**	46.26	72.9	73.36	60.28	51.87	59.51	64.82	51.11	68.81	70.8					
**70**	46.26	72.9	73.36	60.28	51.87	61.28	63.27	50.22	69.47	72.12					
**65**	47.66	71.5	72.9	62.62	51.4	61.95	61.95	49.34	68.81	71.46					
**60**	47.66	71.5	72.9	62.62	51.4	59.96	61.95	50.22	67.26	70.13					
**55**	50.93	74.3	74.77	64.49	51.4	59.73	63.27	50.22	70.58	68.14					
**50**	50.93	74.3	74.77	64.49	51.4	59.73	63.27	49.56	65.49	69.47					
**45**	50.93	74.3	74.77	64.49	51.4	60.62	63.72	49.78	69.47	68.58					
**40**	46.73	66.36	72.9	67.76	46.73	61.5	62.61	48.23	68.36	69.25					
**35**	46.73	66.36	72.9	67.76	46.73	62.17	64.38	47.79	68.14	68.36					
**30**	43.46	63.55	57.01	60.28	35.51	59.07	61.5	45.35	65.93	63.94					
**25**	43.46	63.55	57.01	60.28	35.51	59.29	61.95	44.03	65.93	63.27					
**20**	35.98	54.67	47.2	52.8	35.51	61.5	61.95	46.24	66.15	63.27					
**15**	35.98	54.67	47.2	52.8	35.51	63.05	61.5	52.65	65.04	61.73					
**10**	35.51	35.51	35.51	35.51	35.51	63.05	54.2	52.21	65.04	61.5					
**5**	35.51	35.51	35.51	35.51	35.51	60.18	49.34	47.12	61.5	61.5					

#### State-of-the-art feature selection methods for comparing the performance of the proposed univariate ensemble-based feature selection methodology

In this study, both single-FS methods—namely, IG, gain ratio, symmetric uncertainty, chi-squared, significance, OneR, Relief, ReliefF, and decision rule-based FS (DRB-FS) —and ensemble-based FS methods such as gain-ratio—chi-squared (GR-*χ*^2^), the Borda method, and ensemble-based multifilter FS (EMFFS) method were used as state-of-the-art FS methods for comparing the performance of the proposed uEFS methodology [1, 8, 15, 18, 19, 27, 28]. Each of the FS methods is defined as follows:

IG is an information theoretic as well as a symmetric measure and is one of the popular measures for FS. It is calculated based on a feature’s contribution in enhancing information about the target class label. An equation for IG is given as follows [14]:

$$IG\left(A\right)=Info\left(D\right)-Info_{A}\left(D\right)$$ (1)

where *IG(A)* is the IG of an independent feature or attribute *A*, *Info(D)* is the entropy of the entire dataset, and *Info*_*A*_(*D*) is the conditional entropy of attribute *A* over *D*.

*Gain ratio* is considered to be one of the disparity measures that provides normalized score to enhance the IG result. This measure utilizes the split information value that is given as follows [14]:

$$SplitInfo_{A}\left(D\right)=-\sum_{j=1}^{v}\frac{|D_{j}|}{\left|D\right|}*{\text{log}}_{2}\frac{|D_{j}|}{\left|D\right|}$$ (2)

where *SplitInfo* represents the structure of *v* partitions. Finally, gain ratio is defined as follows [14]:

GainRatio(A)=IG(A)/SplitInfo(A) (3)

*Chi-squared* is a statistic measure that computes the association between the attribute *A* and its class or category *C*_*i*_. It helps to measure the independence of an attribute from its class. It is defined as follows [14]:

$$CHI\left(A,C_{i}\right)=\frac{N*{\left(F_{1}F_{4}-F_{2}F_{3}\right)}^{2}}{\left(F_{1}+F_{3}\right)*\left(F_{2}+F_{4}\right)*\left(F_{1}+F_{2}\right)*\left(F_{3}+F_{4}\right)}$$ (4)

$$CH{\text{I}}_{max}\left(A\right)={\text{max}}_{i}\left(CHI\left(A,C_{i}\right)\right)$$ (5)

where *F*_1_, *F*_1_, *F*_3_, and *F*_4_ represent the frequencies of occurrence of both *A* and *C*_*i*_, *A* without *C*_*i*_, *C*_*i*_ without *A*, and neither *C*_*i*_ nor *A*, respectively, while *N* represents the total number of attributes. A zero value of CHI indicates that both *C*_*i*_ and *A* are independent.

*Symmetric uncertainty* is an information theoretic measure to assess the rating of constructed solutions. It is a symmetric measure and is expressed by the following equation [34]:

$$SU\left(A,B\right)=\frac{2*IG(A|B)}{H(A)+H(B)}$$ (6)

where *IG*(*A*|*B*) represents the IG computed by an independent attribute *A* and the class-attribute *B*. While *H(A)* and *H(B)* represent the entropies of the attributes *A* and *B*.

*Significance* is a real-valued, two-way function used to assess the worth of an attribute with respect to a class attribute [35]. The significance of an attribute *A*_*i*_ is denoted by *σ*(*A*_*i*_), which is computed by the following equation:

$$\sigma \left(A_{i}\right)=\frac{AE\left(A_{i}\right)+CE\left(A_{i}\right)}{2}$$ (7)

where *AE*(*A*_*i*_) represents the cumulative effect of all possible attribute-to-class associations of an attribute *A*_*i*_, which are computed as follows:

$$AE\left(A_{i}\right)=(1/k\sum_{r=1,2,\ldots ,k}{\vartheta_{i}}^{r})-1.0$$ (8)

where *k* represents the different values of the attribute *A*_*i*_.

Similarly, *CE*(*A*_*i*_) captures the effect of change of an attribute value by the changing of a class decision and represents the association between the attribute *A*_*i*_ and various class decisions, which is computed as follows:

$$CE+\left(A_{i}\right)=\left(1/m\right)*(\sum_{j=1,2,\ldots ,m}{A_{i}}^{j})-1.0$$ (9)

where *m* represents the number of classes and + (*A*_*i*_) depicts the class-to-attribute association of the attribute *A*_*i*_.

OneR is the rule-based method to generate a set of rules, which test one particular attribute. The details of this method can be found elsewhere [36].

*Relief* [37] and *ReliefF* [38] are distance-based methods to estimate the weightage of a feature. The original Relief method deals with discrete and continuous attributes; it does not support attempts to deal with incomplete data and is limited to application in two-class problems. ReliefF is an extension of the Relief method that covers the limitations of the Relief method. The details of these methods can be found elsewhere [37, 38].

DRB-FS is a statistical measure to eliminate all irrelevant and redundant features. It allows one to integrate domain-specific definitions of feature relevance, which are based on high, medium, and low correlations that are measured using Pearson’s correlation coefficient, which is computed as follows [29, 39]:

$$r_{XY}=\frac{\sum \left(x_{i}-\bar{x}\right)\left(y_{i}-\bar{y}\right)}{\left(n-1\right)S_{X}S_{Y}}$$ (10)

where $\bar{x}$ and $\bar{y}$ represent the sample means and *S*_*X*_ and *S*_*Y*_ are the sample standard deviations for the features *X* and *Y*, respectively. Here, *n* represents the sample size.

*GR*-*χ*^2^ is an ensemble ranking method that simply adds together the computed ranks of the gain ratio and chi-squared methods [29].

The *Borda method* is a position-based, ensemble-scoring mechanism that aggregates ranking results of features from multiple FS techniques [15]. The final rank of a feature is computed as follows:

$$score_{final}=\sum_{i=1}^{n}score_{pos(i,j)}$$ (11)

where *n* represents the total number of FS techniques and *pos*(*i*, *j*) is the *j*^*th*^ position of a feature ranked by the *i*^*th*^ FS technique.

EMFFS is an ensemble FS method that combines the output of four filter methods—namely, IG, gain ratio, chi-squared, and ReliefF—in order to obtain an optimum selection [18].

#### Statistical measures for evaluating the performance of the proposed univariate ensemble-based feature selection methodology

In this study, precision, recall, f-measure, and the percentage of correct classification were used as evaluation criteria for FS accuracy [8, 12, 15, 18, 29, 40]; second for processing speed; and third as part of a *10-fold cross-validation* technique for computing predictive accuracy to evaluate the performance of machine learning methods or schemes [8, 12, 18, 41–43].

In order to compute the statistical measures (i.e., precision, recall, f-measure, and the percentage of correct classification), the following four measures were required:

- *True positives* (TP) represents the correctly predicted positive values (actual class = yes, predicted class = yes)
- *True negatives* (TN) represents the correctly predicted negative values (actual class = no, predicted class = no)
- *False positives* (FP) represents a contradiction between the actual and predicted classes (actual class = no, predicted class = yes)
- *False negatives* (FN) represents a different contradiction between the actual and predicted classes (actual class = yes, predicted class = no)

Joshi [44] defined these measures as follows:

“*Accuracy* is a ratio of correctly predicted observations to the total observations,” which is computed as follows:

$$Accuracy=\frac{TP+TN}{TP+FP+FN+TN}$$ (12)

“*Precision* is the ratio of correctly predicted positive observations to the total predicted positive observations,” which is computed as follows:

$$Precision=\frac{TP}{TP+FP}$$ (13)

“*Recall* is the ratio of correctly predicted positive observations to all observations in the actual class—yes,” which is computed as follows:

$$Recall=\frac{TP}{TP+FN}$$ (14)

“*F-measure* is the weighted average of Precision and Recall,” which is computed as follows:

$$F-measure=\frac{2*(Recall*Precision)}{\left(Recall+Precision\right)}$$ (15)

## Experimental results of the threshold value selection algorithm

This section demonstrates the results of the proposed TVS algorithm. The purpose is to interpret as well as comment on the results obtained from the experiments.

Table 1 presents the predictive accuracies of eight datasets (i.e., *Cylinder-bands*, *Diabetes*, *Letter*, *Sonar*, *Waveform*, *Vehicle*, *Glass*, and *Arrhythmia*) against five classifiers (*naive Bayes*, *J48*, *kNN*, *JRip*, and *SVM*) with varying threshold values from 100 to 5. In this table, predictive accuracies are recorded as percentages, which were determined by the *10-fold cross-validation* technique, whereas, each threshold value represents the percentage of features retained. After recording the predictive accuracies, the average predictive accuracy of all classifiers as well as datasets against each threshold value was computed, which is shown in Fig 4. This figure depicts the summarized effects of different threshold values on the predictive accuracy of the datasets noted in Table 1.

**Fig 5:**
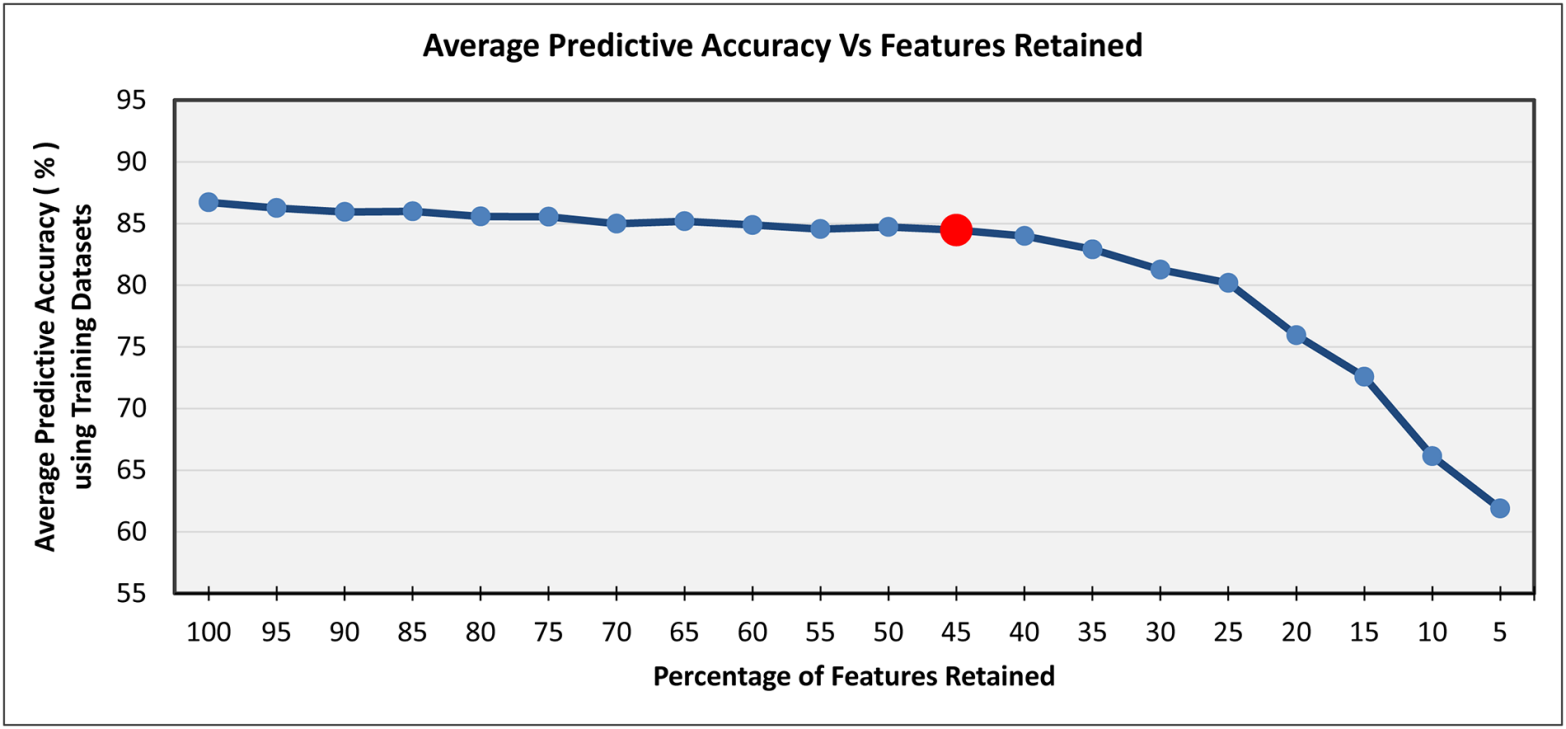
An average predictive accuracy graph using training datasets for threshold value identification.

Furthermore, predictive accuracies using training examples of the aforementioned eight datasets were also recorded against the same five classifiers with varying threshold values from 100 to 5. After recording the predictive accuracies, again, an average predictive accuracy of all classifiers as well as datasets against each threshold value was computed, which is shown in Fig 5.

It can be observed from Figs 4 and 5 that the average predictive accuracy remained consistent from the 100% feature set retained (i.e., no FS) to 45% features retained. After reducing the dataset from 45% retained features to 5% retained features, the predictive accuracy started to decline as well. Therefore, a threshold value of 45 was selected and the top 55% features were chosen. This chunked value (i.e. 45%) was utilized in experimentation for evaluating the uEFS methodology, which provided the best results. This value can also be used to cut off the irrelevant data in future datasets, as this value is also comparable to values obtained in other studies, for example 40% [12, 29] and 50% [45].

## Evaluation of the univariate ensemble-based feature selection methodology

The evaluation phase of any methodology has a key role in investigating the worth of any proposed method. This section covers the experimental setup as well as execution to evaluate the proposed uEFS methodology with state-of-the-art FS methods. The purpose was to check the impact of the proposed methodology on FS suitability in terms of features’ ranking according to the precision, recall, f-measure, and predictive accuracy performance measure factors.

### Experimental setup

For holistic understanding, two studies were performed to evaluate the uEFS methodology by involving nontext and text benchmark datasets. In each study, the methodology was compared with the state-of-the-art FS methods using precision, recall, f-measure, and predictive accuracy performance measure factors. The motivation behind comparing the results achieved with the text and nontext datasets was to check the scalability of the proposed uEFS methodology from small- to high-dimensional data, where *dimension* represents the number of attributes or features.

For the *first study*, eight nontext benchmark datasets of varying complexity (i.e., small to medium size and binary to multiclass problems), were chosen, including *Cylinder-bands*, *Diabetes*, *Letter*, *Sonar*, *Waveform*, *Vehicle*, *Glass*, and *Arrhythmia*, as shown in the Table 2. These datasets were collected from the openML repository available at http://www.openml.org/.

**Table 2:** Selected nontext datasets’ characteristics.

Nontext Dataset	No. of Instances	No. of Attributes	No. of Distinct Classes
Cylinder-bands	540	40	2
Diabetes	768	9	2
Letter	20,000	17	2
Sonar	208	61	2
Waveform	5,000	41	3
Vehicle	846	19	4
Glass	214	10	6
Arrhythmia	452	280	13

For the *second study*, the following four text datasets of varying complexity were selected: *MiniNewsGroups* (http://kdd.ics.uci.edu/databases/20newsgroups/20newsgroups.html), *Course-Cotrain* (http://www.cs.cmu.edu/afs/cs.cmu.edu/project/theo-51/www/co-training/data/course-cotrain-data.tar.gz), *Trec05p-1* (https://plg.uwaterloo.ca/gvcormac/treccorpus/), and *SpamAssassin* (http://csmining.org/index.php/spam-assassin-datasets.html). These datasets are in text form and, to apply the feature-ranking algorithms on these datasets, there is a need to preprocess the text data into a structured form. In order to perform text preprocessing, the following tasks were completed:

1. Remove Hypertext Markup Language tags from web documents, sender as well as receiver information from e-mail documents, URLs, etc.
2. Eliminate pictures and email attachments from the documents
3. Tokenize the documents
4. Remove the noninformative terms like stopwords from the contents
5. Perform the term stemming task
6. Eliminate the low-length terms whose length is less than or equal to 2
7. Finally, generate the feature vectors representing document instances by computing the Term Frequency—Inverse Document Frequency weights.

Table 3 shows the characteristics of the structured form of the text datasets. These datasets also have varying complexity (i.e., small to medium size and binary to multiclass problems).

**Table 3:** Selected text datasets’ characteristics.

Text Dataset	No. of Features	No. of Documents	No. of Distinct Classes
MiniNewsGroups	27,419	1,600	4
Course-Cotrain	13,919	1,051	2
Trec05p-1	12,578	62,499	2
SpamAssassin	9,351	3,000	2

**Fig 6:**
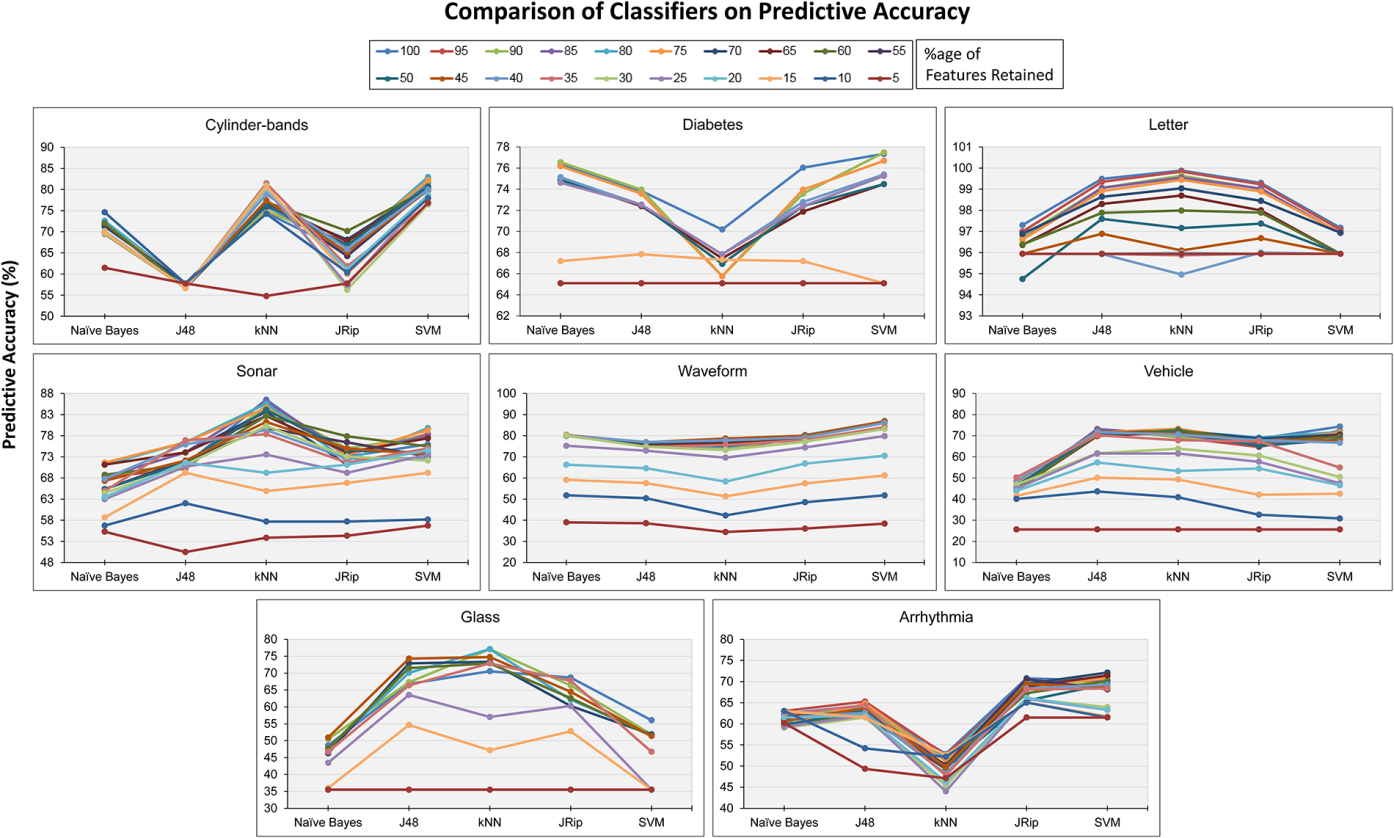
Predictive accuracies of classifiers against benchmark datasets with varying percentages of retained features.

To select a suitable classifier for assessing the proposed uEFS methodology, initially, five well-known classifiers were used: naive Bayes, J48, kNN, JRip, and SVM [8, 12, 15, 18, 29, 40, 45, 46]. Using each classifier, predictive accuracy was measured with a varying percentage of features retained values from 100 to 5, as illustrated in Fig 6. The pictorial results show that, of the five classifiers, SVM and kNN tended to perform best with regard to the above-mentioned datasets. Fig 6 shows the four datasets—namely *Cylinder-bands*, *Diabetes*, *Waveform*, and *Arrhythmia*—on which SVM performed better. Likewise, Fig 6 shows the three datasets (*Letter*, *Sonar*, and *Glass*) on which kNN performed best. In recent years, the SVM classifier has been considered as a dominant tool for dealing with classification problems in a wide range of applications [45] and is largely preferred over other classification methods [46].

Keeping in view with the Fig 6 results and state-of-the-art classifier considerations, finally, the SVM classifier was used to assess the proposed uEFS methodology, as it tends to outperform the F-measures and predictive accuracies for the benchmark datasets [29, 45]. Further, the *SMOreg* function (SVM with sequential minimum optimization) of the SVM classifier was used, which is an improved version of the SVM [47]. Table 4 shows the parameters of the selected classifier.

**Table 4:** Selected classifier parameters.

Classifier	Function	Kernel Type	Epsilon	Tolerance	Exponent	Random Seed
SVM	SMO	Polynomial	1.0E-12	0.001	1	1

For comparison purposes, a standard open-source implementation of this classifier was utilized as provided by the *Waikato Environment for Knowledge Analysis* (WEKA) available at http://weka.sourceforge.net/doc.dev/. Using open-source implementation, a method in Java language was written, which computes precision, recall, f-measure, and predictive accuracy of this classifier using the 10-fold cross-validation technique.

Finally, to compare the computational cost, the performance speed of the proposed methodology as well as state-of-the-art methods were measured on a system having the following specifications:

- Processor: Intel (R) Core (TM) i5-2500 CPU @ 3.30 GHz
- Installed memory (RAM): 16.0 GB
- System type: 64-bit operating system

### Experimental execution

**Fig 7:**
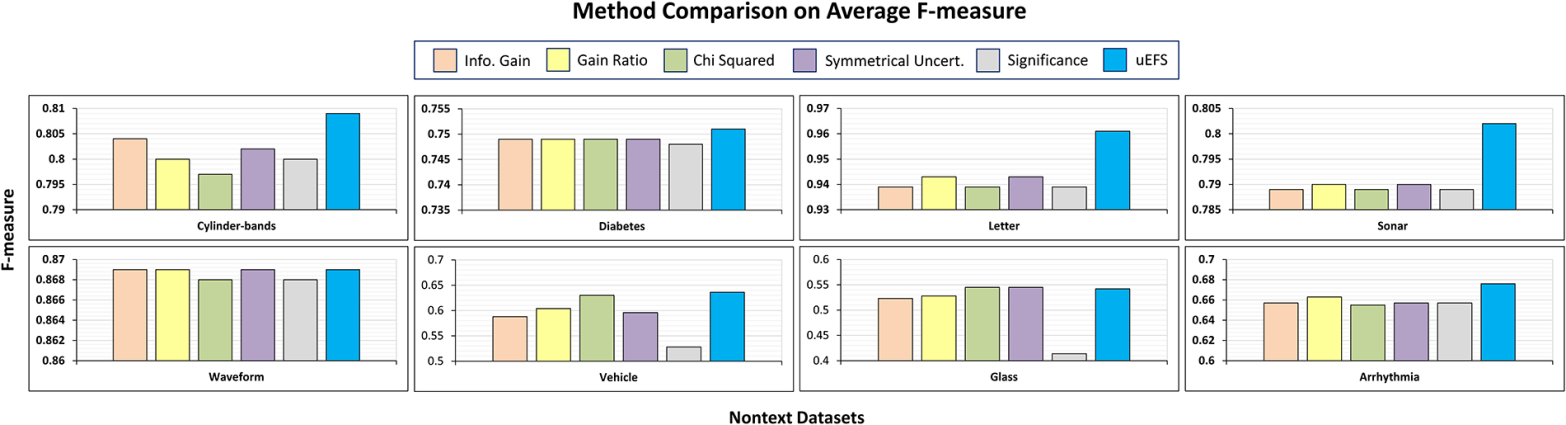
Comparisons of F-measure with existing FS measures.

For the *first study*, a comparison was made between the proposed uEFS methodology and the aforementioned five univariate filter measures, which were used for the proof-of-concept. Fig 7 depicts the difference of the f-measure of the proposed uEFS methodology with each FS measure, which is used in the uEFS methodology. It can be deduced from the results, shown in Fig 7, that the proposed methodology provides competitive results as compared with state-of-the-art FS measures.

For comparison purposes, computed precision and recalls were also used, as recorded in Tables 5 and 6. The results of these two tables also reveal that the proposed methodology provides better results. The proposed uEFS methodology yields significant precision and recall on all nontext datasets except *Glass* against all existing feature selection measures. On recall comparison, the closest competitors to the uEFS methodology were IG, gain ratio, and symmetrical uncertainty measures, which achieved a similar recall of 0.869 with the *Waveform* dataset. Regarding the other datasets, the existing measures achieved a much lower recall as compared with the uEFS. Similarly, with respect to the precision comparison, the chi-squared and symmetrical uncertainty remained the closest competitors to the uEFS for the *Glass* dataset. For the rest of the datasets, the uEFS outperformed the existing FS measures with a significant difference.

**Table 5:** Comparisons of average classifier precision with existing FS measures.

Nontext Dataset	Feature Selection Measures					Proposed Methodology
	IG^a^	GR^b^	CS^c^	SU^d^	S^e^	uEFS
Cylinder-bands	0.805	0.801	0.797	0.803	0.801	**0.811**
Diabetes	0.753	0.753	0.753	0.753	0.738	**0.754**
Letter	0.920	0.962	0.920	0.962	0.920	**0.970**
Sonar	0.789	0.791	0.789	0.791	0.789	**0.803**
Waveform	0.869	0.869	0.868	0.869	0.868	**0.870**
Vehicle	0.586	0.604	**0.642**	0.605	0.534	**0.642**
Glass	0.477	0.484	**0.551**	**0.551**	0.451	0.550
Arrhythmia	0.640	0.647	0.639	0.640	0.639	**0.659**

^a^ IG: information gain,

^b^ GR: gain ratio,

^c^ CS: chi-squared,

^d^ SU: symmetrical uncertainty,

^e^ S: significance

**Table 6:** Comparisons of average classifier recall with existing FS measures.

Nontext Dataset	Feature Selection Measures					Proposed Methodology
	IG^a^	GR^b^	CS^c^	SU^d^	S^e^	uEFS
Cylinder-bands	0.806	0.802	0.798	0.804	0.802	**0.811**
Diabetes	0.759	0.759	0.759	0.759	0.758	**0.760**
Letter	0.959	0.961	0.959	0.961	0.959	**0.970**
Sonar	0.788	0.789	0.788	0.789	0.788	**0.803**
Waveform	**0.869**	**0.869**	0.868	**0.869**	0.868	**0.869**
Vehicle	0.617	0.632	0.655	0.631	0.540	**0.658**
Glass	0.579	0.584	**0.589**	**0.589**	0.481	0.584
Arrhythmia	0.719	0.723	0.717	0.719	0.719	**0.728**

^a^ IG: information gain,

^b^ GR: gain ratio,

^c^ CS: chi-squared,

^d^ SU: symmetrical uncertainty,

^e^ S: significance

A comparison was also made between the predictive accuracies of the uEFS methodology and the five aforementioned univariate filter measures. Table 7 illustrates the comparison of the predictive accuracy of the uEFS methodology with the five FS measures that are used in the uEFS methodology. It can be observed from the Table 7 results that the proposed methodology provides competitive results as compared with existing FS measures. Similarly, it can also be seen from the results shown in Fig 7 and Tables 5, 6, and 7, respectively, that, in terms of f-measure, precision, recall, and predictive accuracy, the proposed methodology did not perform better than existing FS measures on the *Glass* dataset due to having a small size of data, multiple classes, and imbalanced class characteristics.

**Table 7:** Comparisons of predictive accuracy (in %age) of the uEFS with existing FS measures.

Nontext Dataset	Feature Selection Measures					Proposed Methodology	One-Sample T-Test	Paired-Samples T-Test
	IG^a^	GR^b^	CS^c^	SU^d^	S^e^	uEFS	p {Sig. (two-tailed)}	p {Sig. (two-tailed)}
Cylinder-bands	80.56	80.19	79.81	80.37	80.19	**81.11**	**0.002**	**0.029**
Diabetes	75.91	75.91	75.91	75.91	75.89	**76.04**	**0.000**	
Letter	95.94	96.08	95.94	96.08	95.94	**96.97**	**0.000**	
Sonar	78.85	78.86	78.85	78.86	78.85	**80.29**	**0.000**	
Waveform	86.88	86.88	86.86	86.88	86.86	**86.9**	**0.005**	
Vehicle	61.7	63.24	65.48	63.12	54.02	**65.84**	0.093	
Glass	57.94	58.41	**58.88**	**58.88**	48.13	58.41	0.400	
Arrhythmia	71.9	72.35	71.68	71.9	71.9	**72.79**	**0.002**	

^a^ IG: information gain,

^b^ GR: gain ratio,

^c^ CS: chi-squared,

^d^ SU: symmetrical uncertainty,

^e^ S: significance

The result of *one-sample t-test* and *paired-samples t-test* is also illustrated in Table 7. The purpose of performing this test was to determine whether the values obtained from the proposed uEFS methodology were significantly different from the values obtained from existing FS measures. For performing this test against each dataset, FS measures’ values were considered as sample data and the uEFS value was designated as a test value, which is a known or hypothesized population mean. For example, in the case of the *Cylinder-bands* dataset, 81.11 (value generated by the uEFS) was considered to be a test value, while 80.56, 80.19, 79.81, 80.37, and 80.19 (values generated by *IG*, *gain ratio*, *chi-squared*, *symmetrical uncertainty*, and *significance*) were used as sample data. The null hypothesis (*H*_0_) and (two-tailed) alternative hypotheses (*H*_1_) of this test are:

- *H*_0_: 81.11 = $\bar{x}$ (“the mean predictive accuracy of the sample $\bar{x}$ is equal to 81.11”)
- *H*_1_: 81.11 ≠ $\bar{x}$ (“the mean predictive accuracy of the sample $\bar{x}$ is not equal to 81.11”)

In this case, the mean FS measures score for the *Cylinder-bands* dataset (M = 80.22, SD = 0.28) was lower than the normal uEFS score of 81.11, with a statistically significant mean difference of 0.89 (95% confidence interval: 0.54–1.23, t(4) = −7.141, p = .002). Since *p* < .05, we rejected *H*_0_ due to mean predictive accuracy of sample $\bar{x}$ is equal to 81.11 and concluded that the mean predictive accuracy of the sample is significantly different from the existing methodologies’ results. It can be observed from Table 7 that most of the significance (i.e. *p*) values are less than 0.05 (i.e. *p* < .05), which shows that the proposed uEFS methodology results are statistically significantly different from the results of existing methodologies.

Similarly, the *paired-samples t-test* was also performed, to analyze the significance of the proposed methodology. Table 8 reports the paired-samples t-test results. It can be observed also from Table 8 that both of the significance (i.e. *p*) values (one-tailed and two-tailed) are less than 0.05 (i.e. *p* < .05), which shows that the proposed uEFS methodology results are statistically significantly different from existing methodologies result.

**Table 8:** Paired-samples t-test results.

	State-of-the-art Filter-based Measures’ Mean	Proposed uEFS Methodology
Mean	75.970	**77.294**
Variance	164.664	**144.659**
Pearson Correlation	**0.996**	
Hypothesized Mean Difference	0	
df	7	
t Stat	-2.739	
P(T¡ = t) one-tailed	**0.014**	
P(T¡ = t) two-tailed	**0.029**	

For evaluating the computation cost of the proposed FS methodology, the performance speed was also computed, as shown in Table 9. The results indicate that, on average, the proposed methodology takes 0.37 seconds more time than the state-of-the-art filter measures.

**Table 9:** Comparisons of time measure (in seconds) with existing FS measures.

Nontext Dataset	Feature Selection Measures					Proposed Methodology	ATSM^f^	TD^g^	ATD^h^
	IG^a^	GR^b^	CS^c^	SU^d^	S^e^	uEFS	(sec)	(sec)	(sec)
Cylinder-bands	**4.12**	3.28	3.82	3.79	3.59	**4.53**	3.72	0.81	**0.37**
Diabetes	**0.14**	0.11	0.12	0.12	0.12	**0.17**	0.12	0.05	
Letter	4.60	4.12	**4.63**	4.28	4.60	**4.77**	4.45	0.32	
Sonar	0.06	0.05	**0.08**	0.06	0.06	**0.14**	0.06	0.08	
Waveform	1.11	**1.12**	**1.12**	1.09	**1.12**	**2.09**	1.11	0.98	
Vehicle	**0.33**	0.28	0.30	0.28	0.30	**0.39**	0.3	0.09	
Glass	**0.36**	**0.36**	0.33	0.34	0.33	**0.34**	0.34	0	
Arrhythmia	2.67	2.68	2.54	**2.70**	2.64	**3.31**	2.65	0.66	

^a^ IG: information gain,

^b^ GR: gain ratio,

^c^ CS: chi-squared,

^d^ SU: symmetrical uncertainty,

^e^ S: significance,

^f^ ATSM: average time of state-of-the-art measures,

^g^ TD: time difference,

^h^ ATD: average time difference

The proposed FS methodology was also compared with traditional well-known FS methods (i.e., *OneR* and *ReliefF*), as illustrated in Table 10. The results of Table 10 show that the proposed methodology provides competitive results as compared with existing FS methods.

**Table 10:** Comparisons of predictive accuracy (in %age) with existing FS methods.

Nontext Dataset	Feature Selection Methods		Proposed Methodology
	OneR	ReliefF	uEFS
Cylinder-bands	79.63	80.37	**81.11**
Diabetes	75.39	75.52	**76.04**
Letter	**97.14**	96.91	96.97
Sonar	77.88	75.96	**80.29**
Waveform	86.76	**86.90**	**86.90**
Vehicle	64.89	63.83	**65.84**
Glass	49.07	57.01	**58.41**
Arrhythmia	71.02	71.46	**72.79**

Finally, for the *first study*, a comparison of the proposed uEFS methodology with the two state-of-the-art ensemble methods, namely Borda and EMFFS [15, 18], was performed. A methodological comparison of these two methods with the proposed uEFS methodology is illustrated in Table 11. For the proof-of-concept as well as the aforementioned comparisons, five filter measures were used; however, to compare the proposed uEFS methodology with these two state-of-the-art ensemble methods, three [15] and four [18] filter measures defined in each state-of-the-art ensemble method, were used, respectively, as mentioned in Table 11.

**Table 11:** Comparisons of state-of-the-art ensemble methodologies with the proposed uEFS methodology.

State-of-the-art ensemble methodology—I		State-of-the-art ensemble methodology—II	
Borda method [15]	uEFS methodology	EMFFS method [18]	uEFS methodology
1. Consider three filter measures (IG, symmetric uncertainty, chi-squared)	1. Consider three filter measures (IG, symmetric uncertainty, chi-squared)	1. Consider four filter measures (IG, gain ratio, chi-squared, ReliefF)	1. Consider four filter measures (IG, gain ratio, chi-squared, ReliefF)
2. Compute the ranks using each filter measure	2. Compute the ranks using each filter measure	2. Compute the ranks using each filter measure	2. Compute the ranks using each filter measure
3. Sort the computed ranks in an ascending order	3. Compute the scaled ranks of each computed ranks	3. Sort the computed ranks in an ascending order	3. Compute the scaled ranks of each of the computed ranks
4. Assign a score to each feature in a list based on its position	4. Compute the combined sum of all computed ranks	4. Select the top one-third split of each filter measure’s output	4. Compute the combined sum of all computed ranks
5. Compute the sum of all the positional scores from all the lists	5. For each feature, compute the combined rank by adding all computed scaled ranks	5. Define the feature count threshold	5. For each feature, compute the combined rank by adding all computed scaled ranks
6. Sort the computed sum in an ascending order to generate the final ranked feature set	6. Sort the list in an ascending order after computing the score, weight, and priority of each feature	6. Compute the feature occurrence rate among the filter measures	6. Sort the list in an ascending order after computing the score, weight, and priority of each feature
		7. If the feature count is less than the threshold, drop the feature; otherwise, select the feature	7. Determine the threshold value using the proposed TVS method
			8. Apply the threshold value to drop the irrelevant features and to select the final ranked feature set

After applying the ensemble-based Borda and EMFFS methods, the predictive accuracy and F-measures of the proposed uEFS methodology, using three and four filter measures, respectively, were computed, as shown in Tables 12 and 13. The results of Tables 12 and 13 reveal that the proposed methodology provides better results as compared with the two state-of-the-art ensemble methods [15, 18]. It can be observed from the results shown in Tables 12 and 13 that, in terms of predictive accuracy and f-measure, the performance of the proposed methodology is the same as the state-of-the-art ensemble methods regarding the *Letter* dataset, while the proposed methodology did not perform better than the EMFFS method for the *Arrhythmia* dataset due to having a small size of data, multiple classes, and imbalanced class characteristics.

**Table 12:** Comparisons of predictive accuracy and F-measure with the Borda method [15].

Nontext Dataset	Predictive Accuracy (%)		F-measure	
	Borda method [15]	uEFS (three filter measures)	Borda method [15]	uEFS (three filter measures)
Cylinder-bands	57.78	**80.37**	0.423	**0.802**
Diabetes	65.10	**75.91**	0.513	**0.749**
Letter	**95.94**	**95.94**	**0.939**	**0.939**
Sonar	66.83	**78.85**	0.667	**0.789**
Waveform	31.80	**86.88**	0.311	**0.869**
Vehicle	59.22	**63.12**	0.58	**0.596**
Glass	40.19	**58.88**	0.316	**0.545**
Arrhythmia	64.60	**71.90**	0.564	**0.657**

**Table 13:** Comparisons of predictive accuracy and F-measure with the EMFFS method [18].

Nontext Dataset	Predictive Accuracy (%)		F-measure	
	EMFFS method [18]	uEFS (four filter measures)	EMFFS method [18]	uEFS (four filter measures)
Cylinder-bands	80.74	**81.48**	0.805	**0.813**
Diabetes	75.52	**75.91**	0.739	**0.749**
Letter	**95.94**	**95.94**	**0.939**	**0.939**
Sonar	78.37	**80.29**	0.784	**0.803**
Waveform	86.48	**86.90**	0.864	**0.869**
Vehicle	41.73	**63.12**	0.392	**0.596**
Glass	54.67	**58.88**	0.491	**0.545**
Arrhythmia	**73.23**	71.68	**0.672**	0.658

**Fig 8:**
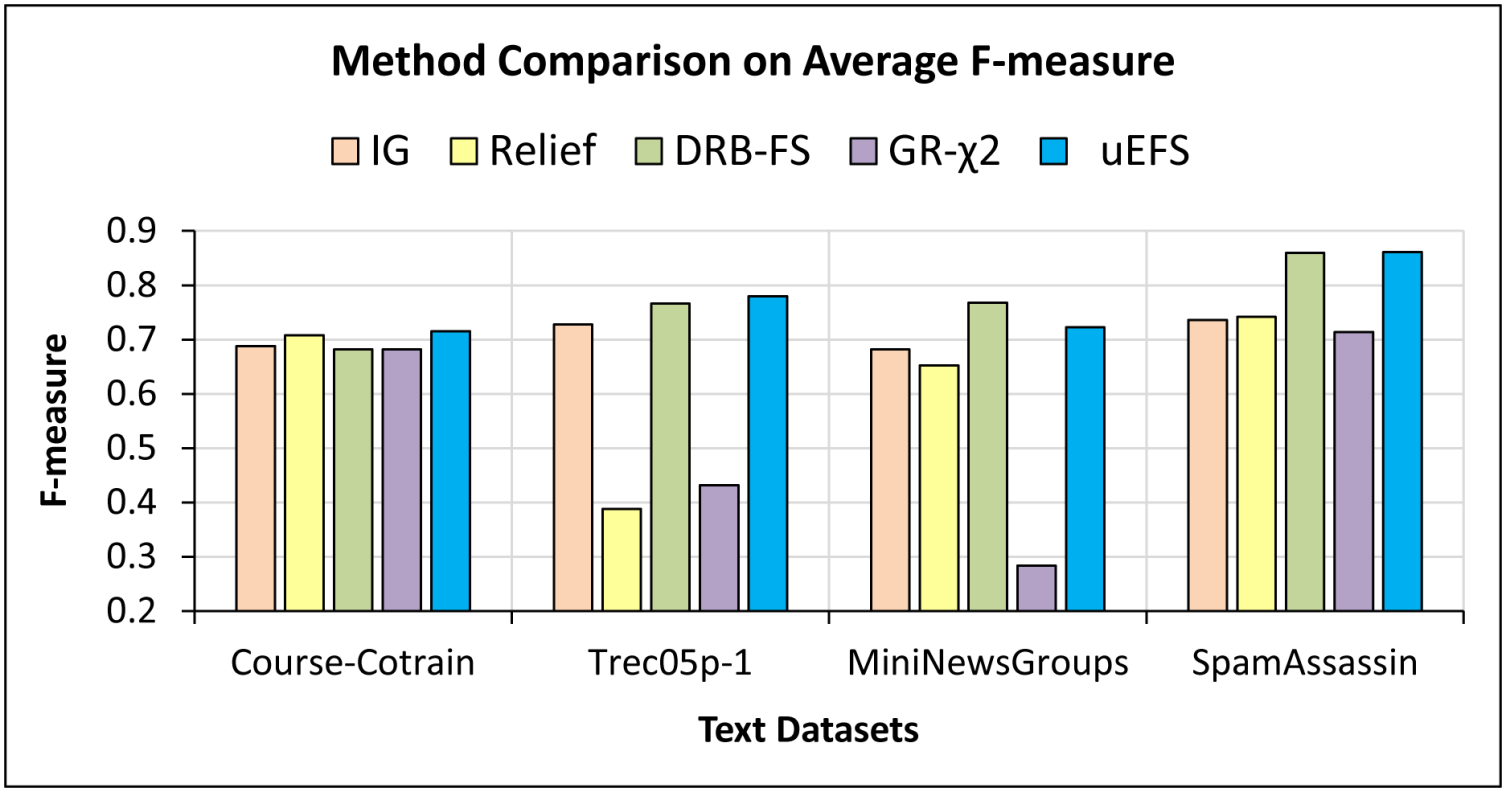
Comparisons of F-measure with existing FS measures [29, 37, 39, 48].

**Fig 9:**
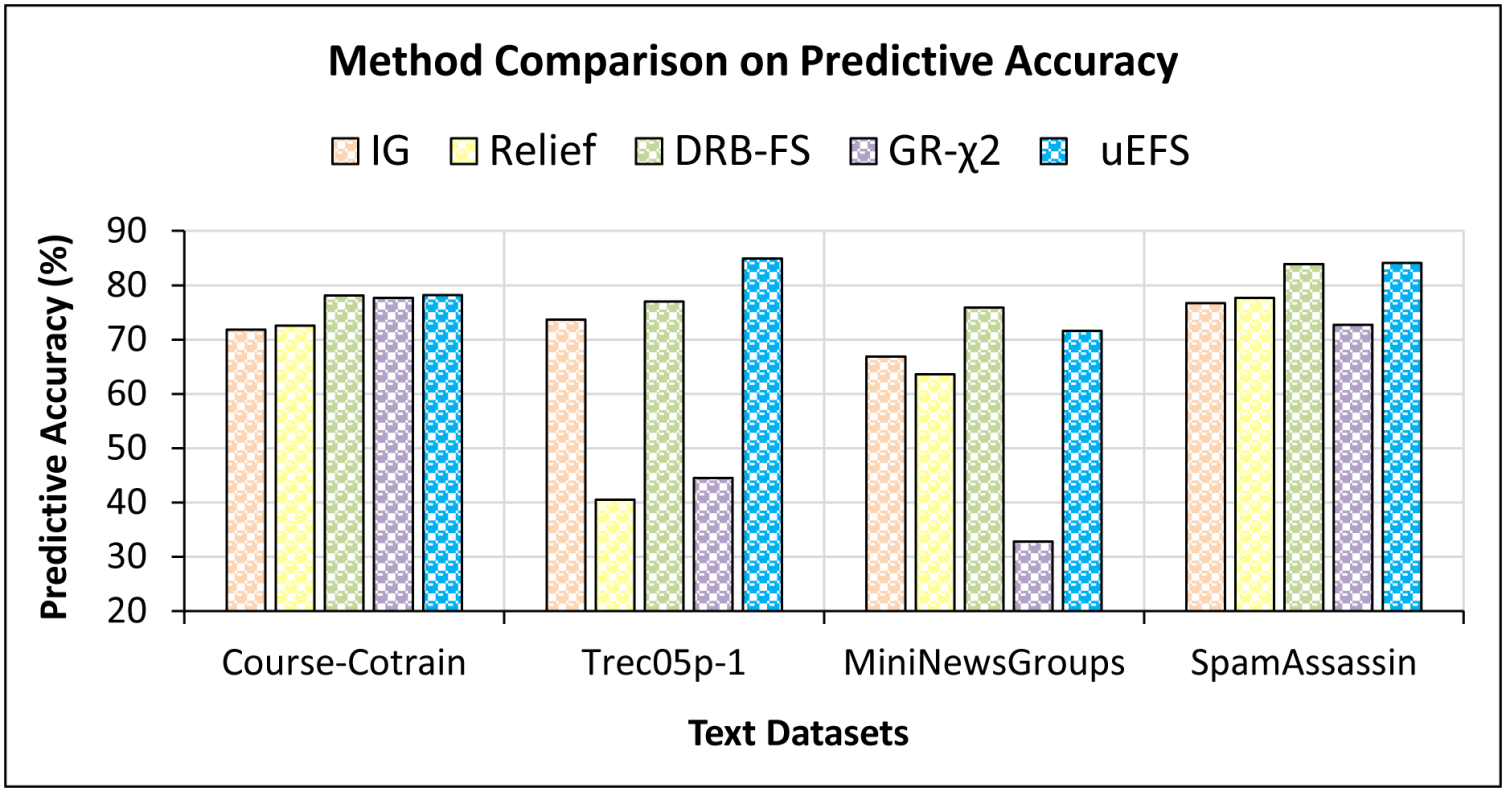
Comparisons of predictive accuracy with existing FS measures [29, 37, 39, 48].

For the *second study*, a comparison of the proposed uEFS methodology with state-of-the-art FS methodologies was performed. The proposed methodology outperforms most of the existing algorithms and individual FS measures in terms of f-measure as well as predictive accuracy. It can be observed from Figs 8 and 9 that the average f-measure and predictive accuracy results of the proposed uEFS methodology on multiple text datasets are higher than existing techniques.

On the other hand, the individual numeric values of precision against each dataset are shown in Table 14. For the *SpamAssassin* benchmark dataset, the uEFS outperformed the existing algorithms with a precision of 0.858. Similarly, the uEFS achieved an average of 0.669 precision for the *Course-Cotrain* data which is close enough to the *Relief* algorithm with a difference of 0.004, which achieved the highest precision against the existing algorithms. On the other hand, while comparing the average classifier recall, shown in Table 15, it was noticed that the proposed uEFS methodology outperforms all of the existing algorithms with a recall of 0.850 and 0.864 for the *Trec05p-1* and *SpamAssassin* benchmarks, respectively.

**Table 14:** Comparisons of average classifier precision with existing FS methods [29, 37, 39, 48].

Text Dataset	Feature Selection Algorithms				Proposed Methodology
	IG	Relief	DRB-FS	GR-*χ*^2^	uEFS
Course-Cotrain	0.668	**0.673**	0.609	0.648	0.669
Trec05p-1	0.836	0.375	**0.839**	0.423	0.721
MiniNewsGroups	0.730	0.708	**0.811**	0.272	0.764
SpamAssassin	0.708	0.710	0.857	0.701	**0.858**

**Table 15:** Comparisons of average classifier recall with existing FS methods [29, 37, 39, 48].

Text Dataset	Feature Selection Algorithms				Proposed Methodology
	IG	Relief	DRB-FS	GR-*χ*^2^	uEFS
Course-Cotrain	0.717	0.711	**0.780**	0.776	0.768
Trec05p-1	0.731	0.410	0.764	0.451	**0.850**
MiniNewsGroups	0.669	0.636	**0.759**	0.327	0.686
SpamAssassin	0.766	0.778	0.863	0.727	**0.864**

It can also be observed from the results, shown in Tables 14 and 15 that, in terms of precision and recall, the proposed methodology did not perform better than the DRB-FS measure for some datasets due to considering only those measures in terms of proof-of-concept purposes, which measure only relevancy and ignore the feature redundancy factor. As the DRB-FS measure eliminates all irrelevant as well as redundant features and is also based on predefined domain-specific definitions of feature relevance [29, 39], there is a chance that the DRB-FS can produce better results as compared with the proposed methodology. However, in terms of f-measure, which is the weighted average of precision and recall, overall, the proposed methodology performs better than the DRB-FS measure as shown in Fig 8.

The uEFS methodology was evaluated rigorously with respect to text and nontext benchmark datasets having small- to high-dimensional data size and provides competitive results as compared with state-of-the-art FS methods, which indicates that our proposed ensemble approach is more robust across text and nontext datasets. The above-mentioned results also provide evidence that the uEFS methodology is stable towards producing a similar and most likely higher degree of predictive accuracy and f-measure value across a wide variety of datasets.

## Conclusions and future directions

FS is an active area of research for the data mining and text mining research community. In this study, we introduce an efficient and comprehensive uEFS methodology to select informative features from a given dataset. For the uEFS methodology, we first proposed an innovative UFS algorithm to generate a final-ranked list of features without the use of any learning algorithm, high computational cost, and any individual statistical biases of state-of-the-art feature-ranking methods. For defining a cutoff point to remove irrelevant features, we then proposed a TVS algorithm. An extensive experiment was performed to evaluate the uEFS methodology using standard benchmark datasets; the results show that the uEFS methodology provides competitive accuracy as compared with state-of-the-art methods. The proposed uEFS methodology contributes to FS, which is a key step in decision support systems. It can be utilized in real-world applications such as DDKAT [19] to assist the domain expert in selecting informative features for generating production rules from a dataset, or extracting relative information from open data for constructing reliable domain knowledge. The current version of the UFS code and its documentation are freely available and can be downloaded from the GitHub open-source platform [20, 21].

Currently, the proposed methodology incorporates state-of-the-art univariate filter measures to consider the relevance aspect of feature ranking and ignores the features’ redundancy aspect. In the future, we will extend our methodology for incorporating multivariate measures to consider the redundancy aspect of feature subset selection. Similarly, the proposed methodology does not evaluate the suitability of a measure or its precision. In order to consider that factor, we will also investigate the application of fuzzy logic for determining the cutoff threshold value in the future. Lastly, the proposed methodology was applied to text and nontext benchmark datasets to evaluate the model performance. In the future, we will experiment with our proposed uEFS methodology on other application domains such as microarray datasets to check the goodness on all applications. Above all, we also intend to integrate our proposed methodology into another research project, called Intelligent Medical Platform (IMP) available at http://imprc.cafe24.com/.
